# Developing an eyeball positioning method in the eye orbit for craniofacial identification in Korean population

**DOI:** 10.1038/s41598-024-66833-0

**Published:** 2024-07-11

**Authors:** Joon Yeol Ryu, Yeon-Kyung Park, Ji-Hwan Park, Jeong Uk Seo, Byung-Yoon Roh, Eui-Joo Kim, Chang-Un Choi, Kyoung Min Koh, Won-Joon Lee

**Affiliations:** 1 Division of Forensic Medicine, National Forensic Service, Wonju, 26460 Republic of Korea; 2 Department of Forensic Medicine, National Forensic Service Gwangju Institute, Gwangju, 57248 Republic of Korea; 3 Department of Forensic Medicine, National Forensic Service Seoul Institute, Seoul, 08063 Republic of Korea

**Keywords:** Anatomy, Anthropology

## Abstract

We analysed the skulls and faces of Korean subjects using anthropometric methods to understand the anatomical characteristics of the eyeball and eye orbit region of Korean population and to determine the correlations between the hard and soft tissues around the eyeball and eye orbit region. In total, 82 sections in the region were measured to determine the correlations; among them, 34 showed significant differences by sex, and 6 showed significant differences by age. As the distance from the centre of the eye lens to the eye orbit is calculated as a ratio, we determined that the centre of the eye lens is located relatively on the lateral and superior position in each eye orbit in front view. Fourteen sections that could be used for craniofacial reconstruction/approximation in men and women were selected. Regression equations were derived according to the correlation of each section, and their reliabilities were verified by out of sample validation tests. Therefore, our results increase the accuracy of eyeball position determination, which would be useful for more efficient craniofacial reconstruction/approximation of the Korean population and should improve the efficiency of facial recognition.

## Introduction

Craniofacial reconstruction/approximation is a face recreation tool in the craniofacial identification that is used in forensic investigations to identify unknown skulls^1^. This tool is mostly applied after the failure of other forensic identification methods, such as fingerprints, DNA or teeth by National Forensic Service (NFS) in South Korea.

The created face image is used for forensic identification and recognition by other people, particularly friends, family members and relatives of the person whose face was recreated. Hence, it is crucial to estimate and predict the size and shape of the facial features and their relative positions in the recreated face for face recognition.

In the process of facial recognition, visual fixations occur at various locations on the face with differing frequencies^2,3^. Previous studies have consistently identified the regions surrounding the eyes, nose, and mouth as the most significant facial features for identification, drawing focus for fixations in these areas^4–6^. Among these features, the eyes hold particular prominence^]^. Consequently, accurate prediction of the size and position of the eyeballs within a reconstructed face significantly impacts the efficacy of facial recognition.

We investigated the anatomical features of the eyeball and eye orbit to develop a method to estimate the eyeball position via anthropometric correlation analysis of the eyeball, orbit, and entire skull in the Korean population.

Conventional eyeball placement methods used for craniofacial reconstruction/approximation at the NFS relied on the research on ethnic groups other than the Korean population^10^. Considering the predominantly homogeneous nature of the Korean population, a method based on Koreans is needed.

To achieve this, we further developed facial landmarks and reference planes using a standardized approach, building upon previous studies that investigated correlations between eyebrow/orbit positioning and the nose/nasal aperture groove^11,12^. Regression equations were developed to estimate eye position and protrusion in the eye orbit; these data were combined with other anatomical features around the eyes to reconstruct Korean features. The appropriate proportions of, and distances between, facial features were estimated to improve the correlations among features. This process increases the effectiveness of facial recognition, which would also help forensic identification.

## Methods

### Subject selection

This research was conducted using 171 postmortem computed tomography (PMCT) data of autopsied Korean subjects at the NFS Seoul Institute between 2018 and 2020. The subjects included 130 males and 41 females with a mean age of 44 years (range 20–83 years) (Table 1). All the subjects CT scanned within 48 h of postmortem. Subjects with recognizable changes in the morphology of the head or face due to illness or the cause of death were excluded, as were subjects with congenital malformations or prosthetics in craniofacial region.

**Table 1:** Subject classification by age and sex.

Age	Number		
	Male	Female	Total
20–39	49	23	72
40–59	55	14	69
60–83	26	4	30
Total	130	41	171

### Post-mortem computed tomography

The subjects were scanned at a tube voltage of 120 kV, tube current of 170 mA, and a 0.3 mm slice increment using a SOMATOM Definition AS + system (Siemens Healthineers, Erlangen, Germany). Whole-body 2500 DICOM axial images were obtained. Only the head was selected for the detailed images, and the images were exported without any biological or personal information, except age and sex. Each dataset consisted of 750–900 Digital Imaging and Communications in Medicine (DICOM) files.

### Converting the CT data

The PMCT data were converted to three-dimensional (3D) models in 3D visualization and analysis software, Materialise Mimics. The skull, eyeballs and eye lens were separated from each other by adjusting the Hounsfield Units and converted into 3D STL files.

### Measurement selection

We used 20 landmarks (Supplementary Table A) and 43 planes positioned in the skull and head of the subject for the facial measurements. 43 planes were generated around the 3 anatomical reference planes (Medial sagittal plane, Orbitale transverse plane, Coronal plane, Table 2). Each measurement was the perpendicular distance between a landmark and a reference plane, which is basically the same as the previous studies^11,12^.

**Table 2:** Definitions of the landmarks and reference planes.

	Definition
Reference plane	
Median sagittal plane	Plane passing through 3 landmarks, Nasion, prosthion, auriculare midpoint
Orbitale transverse plane	Plane passing through 2 landmarks, orbitale left and auriculare midpoint and orthogonal to the medial sagittal plane
Coronal plane	Plane passing through 1 landmark, bregma and orthogonal to the median sagittal and orbitale transverse planes
Landmark	
Nasion	The junction of the internasal and nasofrontal suture
Prosthion	Median point between the central incisors on the anterior most margin of the maxillary alveolar rim
Auriculare	The innermost point around the external auditory meatus leading from the zygomatic process
Auriculare midpoint	The midpoint of the left & right auriculares
Orbitale left	The lowest point on the left orbital rim
Bregma	The junction of sagittal and coronal suture

**Figure 1:**
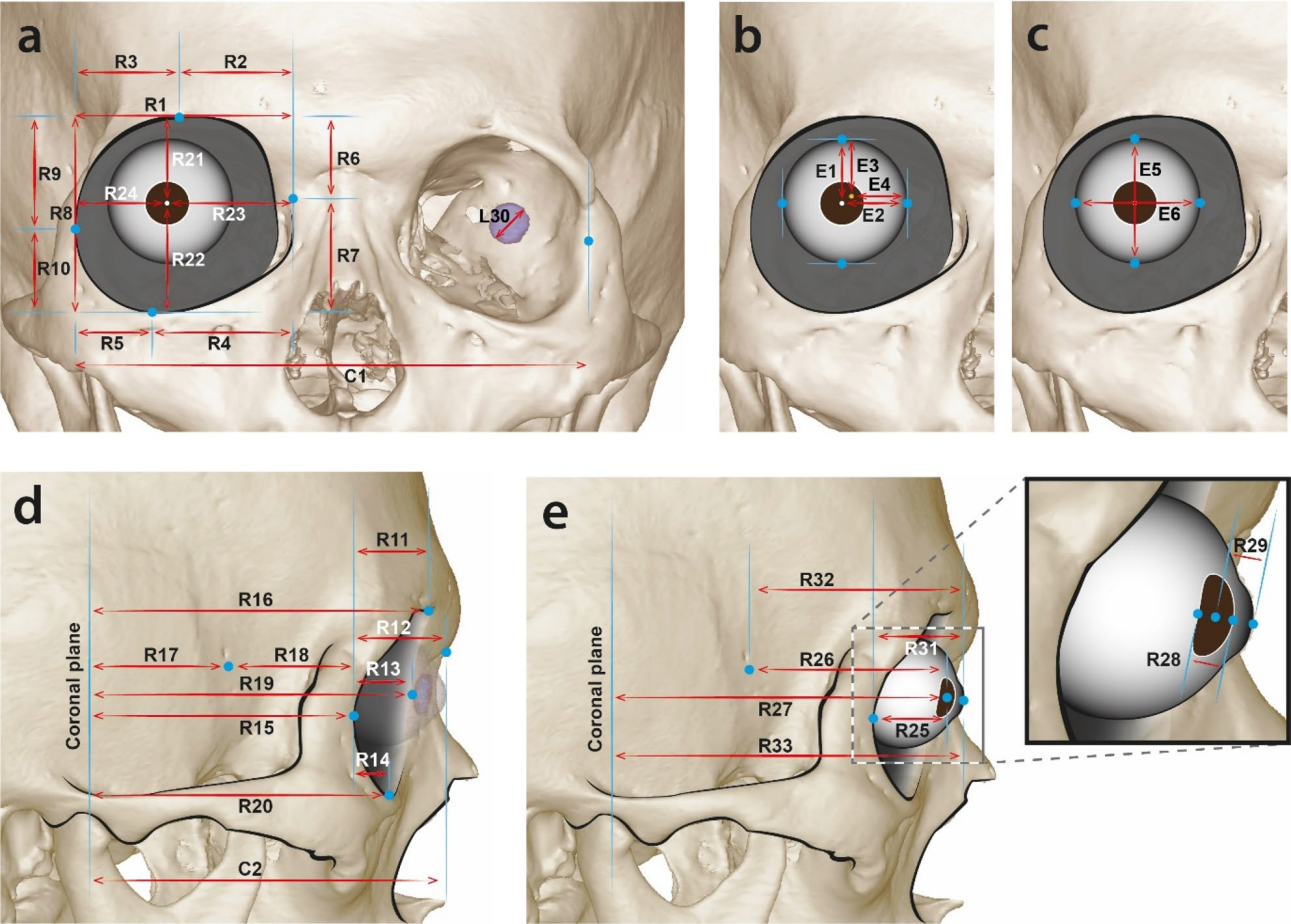
Measurement sections in anterior view (**a**). Distances to the centre of the eyeball and eye lens centre from the farmost medial and superior edge in anterior view (**b**). Eyeball thicknesses in horizontal and longitudinal directions (**c**). Measurement sections in lateral view (**d**,**e**). R represents the right side. C is bilateral symmetry. The sections in which left-side (L) measurements were made are identical to those of the right side.

In total, 82 distances, which covered the orbit and eyeball region and a fairly large portion of the skull and head, were measured (see Fig. 1, Table 3). The measurements in front view were selected to examine the positions of the eyeballs and eye lens in the orbit and they were selected in lateral view to calculate the protrusion of the eyeball.

**Table 3:** Measurement sections used in this study.

Measurement section		Direction
Midline (central)		
C1	Lateral orbit left (landmark)—Lateral orbit right (sagittal plane)	Distance
C2	Nasion (landmark)—Coronal plane	Depth
Bilateral		
1	Lateral orbit (landmark)—Medial orbit (sagittal plane)	Distance
2	Supraorbitale (landmark)—Medial orbit (sagittal plane)	Distance
3	Supraorbitale (landmark)—Lateral orbit (sagittal plane)	Distance
4	Orbitale (landmark)—Medial orbit (sagittal plane)	Distance
5	Orbitale (landmark)—Lateral orbit (sagittal plane)	Distance
6	Medial Orbit (landmark)—Supraorbitale (transverse plane)	Height
7	Medial orbit (landmark)—Orbitale (transverse plane)	Height
8	Supraorbitale (landmark)—Orbitale (transverse plane)	Height
9	Lateral orbit (landmark)—Supraorbitale (transverse plane)	Height
10	Lateral orbit (landmark)—Orbitale (transverse plane)	Height
11	Supraorbitale (landmark)—Lateral orbit (coronal plane)	Depth
12	Nasion (landmark)—Lateral orbit (coronal plane)	Depth
13	Medial orbit (landmark)—Lateral orbit (coronal plane)	Depth
14	Orbitale (landmark)—Lateral orbit (coronal plane)	Depth
15	Lateral orbit (landmark)—Coronal plane	Depth
16	Supraorbitale (landmark)—Coronal plane	Depth
17	Optic canal point (landmark)—Coronal plane	Depth
18	Optic canal point (landmark)—Lateral orbit (coronal plane)	Depth
19	Medial orbit (landmark)—Coronal plane	Depth
20	Orbitale (landmark)—Coronal plane	Depth
21	Lens centre (landmark)—Supraorbitale (transverse plane)	Height
22	Lens centre (landmark)—Orbitale (transverse plane)	Height
23	Lens centre (landmark)—Medial orbit (sagittal plane)	Distance
24	Lens centre (landmark)—Lateral orbit (sagittal plane)	Distance
25	Lens centre (landmark)—Lateral orbit (coronal plane)	Depth
26	Lens centre (landmark)—Optic canal point	Depth
27	Lens centre (landmark)—Coronal plane	Depth
28	Lens anterior (landmark)—Lens posterior	Depth
29	Cornea (landmark)—Lens centre (coronal plane)	Depth
30	Lens	Diameter
31	Cornea (landmark)—Lateral orbit (coronal plane) (25 + 29)	Depth
32	Cornea (landmark)—Optic canal point (26 + 29)	Depth
33	Cornea (landmark)—Coronal plane (27 + 29)	Depth
Index		
Section 1/section 8		Ratio
Eyeball		
E1	Lens centre—Globe superior (transverse plane)	Distance
E2	Lens centre—Globe Medial (sagittal plane)	Distance
E3	Globe centre—Globe superior	Distance
E4	Globe centre—Globe medial	Distance
E5	Globe superior—Globe inferior	Distance
E6	Globe lateral—Globe medial	Distance

### Statistical analysis

All data were analysed using SPSS ver. 17.0 (SPSS, Chicago, IL, USA). Correlations between skull and eyeball/eye lens was detected using Pearson correlation analysis. Simple linear regression was used to analyse measurement sections. Regression equations, which are applicable to the craniofacial reconstruction/approximation were developed. Independent sample *t*-test, paired *t*-test and ANOVA were employed to detect the differences in features according to sex, age, and bilateral relationship in the face and skull. Technical error of measurement (TEM) was employed to see the anthropometric measure imprecision assessing inter- and intra-observer repeatability. The calculation of absolute TEM was square root of measurement error variance referring Ulijaszek, S. J. & Kerr, D. A^13^.

### Out of sample validation

To evaluate the reliability and precision of our developed prediction methods, the same measurement sections of the 30 subjects out of from our samples were measured. T-test was employed to compare the measurement values and prediction values.

### Ethics declarations

All experimental protocols performed in this study were approved by the Institutional Review Board (IRB) of the National Forensic Service (IRB approval number: 906-210415-BR-003-01). All procedures and methodology performed for this study involving human subjects were in accordance with the guidelines and regulations of the institutional research committee and with the 1964 Helsinki Declaration and its later amendments. The need for informed consent from the next of kin was waived because all autopsy procedures at the NFS Seoul Institute are performed under a court-approved warrant. The IRB of NFS approval for a waiver of written informed consent was also obtained.

## Results

### Technical error of measurement analysis

The majority of the TEMs in the measurement sections are deemed acceptable. From the TEM results, 7 measurement sections in the intra-observer TEM and 12 measurement sections in the inter-observer TEM slightly exceeded the acceptable range, and these sections have been excluded from the prediction methods (Supplementary material (TEM)).

### Data analysis by age and sex of the subjects

**Figure 2:**
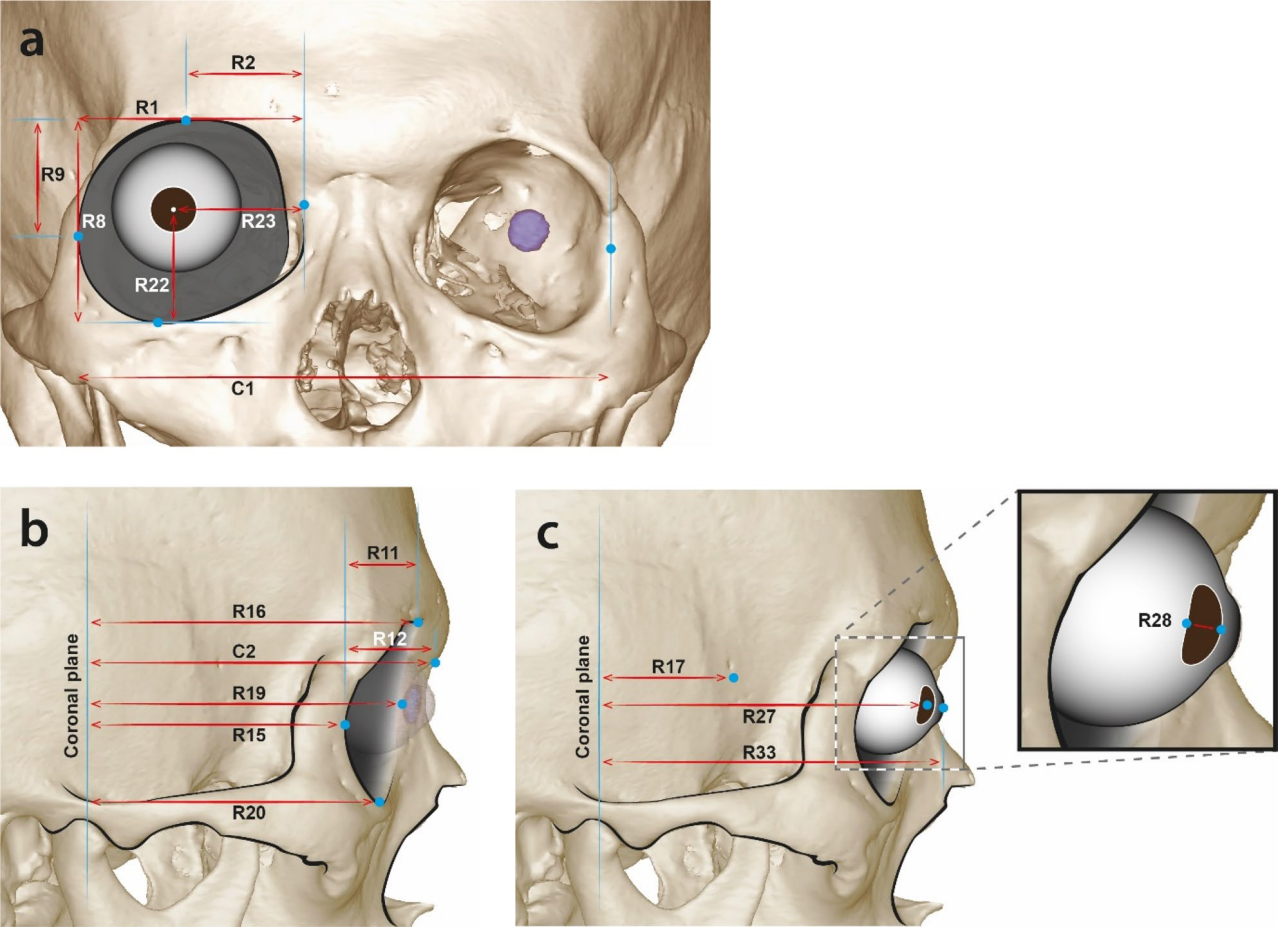
Orbit and eyeball measurement values that significantly differed by sex. R represents the right side. C is bilateral symmetry. The left-side measurement sections are the same as those of the right side.

Significant differences in eyeball vertical diameter [LE(Left Eyeball)5, RE(Right Eyeball)5], vertical position of the eye lens [L(Left)21, R(Right)21] and the eye lens thickness [L(Left)28, R(Right)28] were observed between the age groups. The eyeball vertical diameter and vertical position of the eye lens decreased, and the eye lens thickness increased by aging. There were also significant differences by sex in various sections (see Fig. 2, Table 4). Most values were greater in males.

**Table 4:** Mean (M) and standard deviation (SD) of the measurements by sex.

Measurement section	Male (mm)			Female (mm)		t	P
	M		SD	M	SD		
C1	101.37		3.47	96.60	3.36	7.725	0.000***
C2	76.21		6.04	70.57	5.33	5.356	0.000***
1	L	40.68	1.60	38.96	1.65	5.951	0.000***
	R	40.72	1.58	39.24	1.60	5.222	0.000***
2	L	16.18	2.45	15.14	2.33	2.396	0.018*
	R	16.10	2.53	15.00	2.38	2.453	0.015*
8	L	36.80	2.19	35.72	1.56	3.487	0.001**
	R	36.72	1.97	35.60	1.58	3.338	0.001**
9	L	21.25	3.66	19.59	3.07	2.872	0.005**
	R	20.96	3.65	19.40	3.03	2.49	0.014*
11	L	11.19	1.93	10.22	1.92	2.828	0.005**
	R	11.59	2.02	10.41	1.98	3.273	0.001**
12	L	16.38	2.30	15.13	2.30	3.03	0.003**
	R	16.76	2.71	15.24	2.27	3.265	0.001**
15	L	59.83	5.91	55.44	5.36	4.238	0.000***
	R	59.39	6.16	55.14	5.80	3.901	0.000***
16	L	71.02	5.55	65.65	4.80	5.569	0.000***
	R	70.97	5.61	65.55	5.27	5.474	0.000***
17	L	25.25	7.10	25.02	6.86	3.327	0.001**
	R	21.19	5.86	20.99	6.40	3.334	0.001**
19	L	70.02	5.99	65.40	5.20	4.442	0.000***
	R	69.94	6.07	65.30	5.42	4.375	0.000***
20	L	66.35	6.48	62.01	5.43	3.477	0.000***
	R	66.32	6.80	61.83	6.05	3.784	0.000***
22	L	19.83	1.43	18.94	1.46	3.477	0.001**
	R	20.02	1.38	19.04	1.49	3.898	0.000***
23	L	23.12	1.88	21.95	1.66	3.545	0.001**
	R	23.20	1.76	22.13	1.45	3.546	0.001**
27	L	70.72	6.51	65.40	5.98	4.647	0.000***
	R	70.50	6.65	65.27	6.10	4.481	0.000***
28	L	4.05	0.57	3.83	0.58	2.111	0.036*
	R	4.10	0.59	3.85	0.65	2.313	0.022*
33	L	75.78	6.39	71.04	6.05	3.442	0.001**
	R	75.63	6.60	70.46	6.45	3.604	0.000***

R represents the right side. C is bilateral symmetry. The sections in which left-side measurements were made are identical to those of the right side. *p < 0.05, **p < 0.01, ***p < 0.001.

In the front view, the vertical diameter of the eyeball was 23.68 ± 1.18 mm in males and 23.42 ± 0.73 mm in females; the horizontal diameter was 23.64 ± 1.14 mm in males and 23.45 ± 1.17 mm in females. The male eyeball was slightly larger in both directions, but the difference was not statistically significant.

### Location of the eye lens

**Figure 3:**
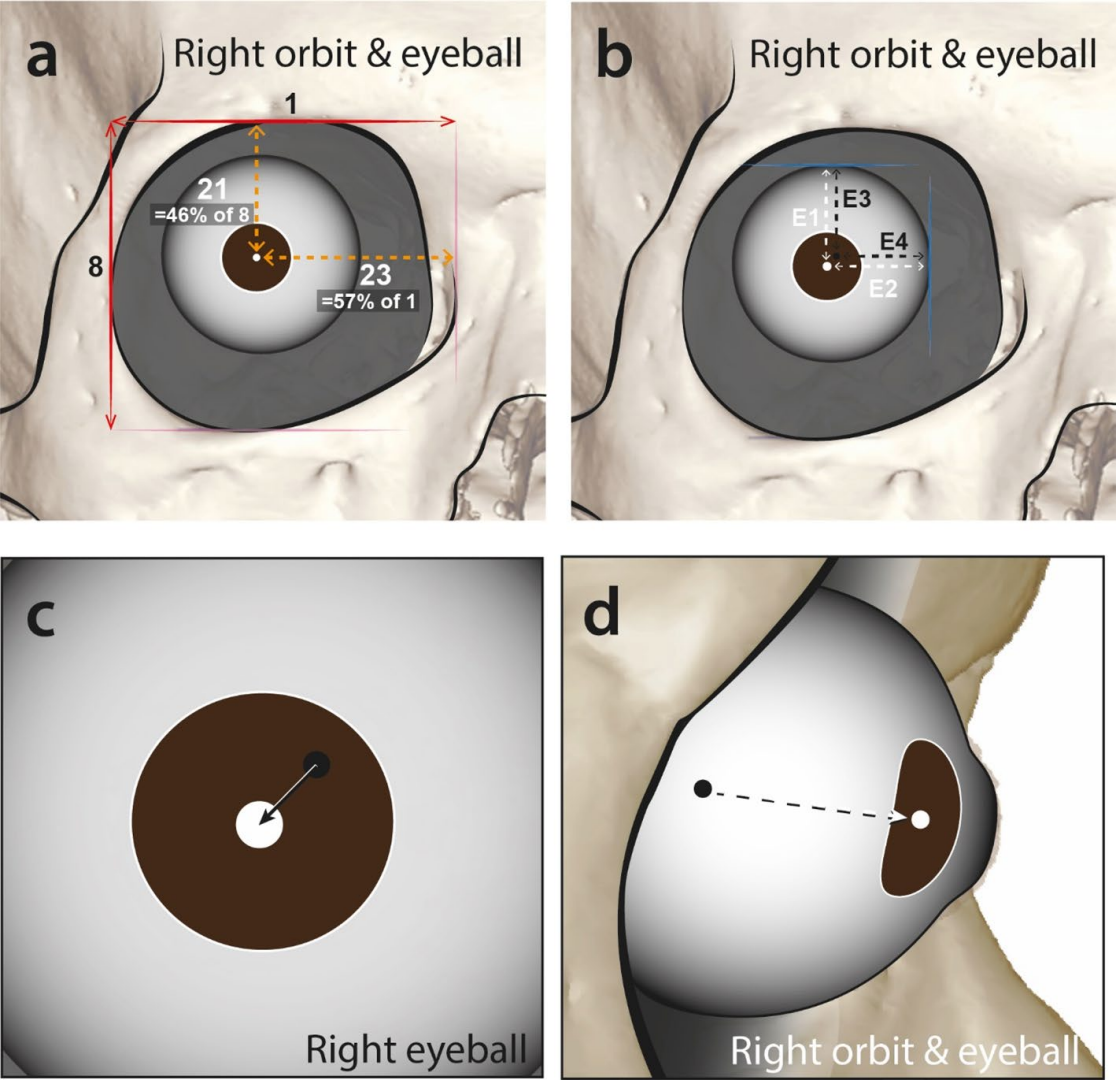
Proportional position of the centre of eye lens to the width and height of the orbit (**a**). Position of the eyeball & eye lens. E1 and E2 are the eye lens centre coordinates and E3 and E4 are the eyeball centre coordinates in anterior view (**b**). The relative position of the centre of eyeball and eye lens, anterior view (**c**) and lateral view (**d**).

The distance from the topmost point of the eye orbit to the eye lens centre (section #21) was 16.83 ± 1.58 mm in males and 16.67 ± 1.25 mm in females; the innermost medial point of the eye orbit to the centre of eye lens (section #23) was 23.16 ± 1.82 mm in males and 22.04 ± 1.56 mm in females. Over all measurements regardless of sex, the distance from the medial eye orbit (the innermost point of orbit) to the centre of eye lens average 57% of the eye orbit width; that from the supraorbitale (the topmost point of orbit) to the centre of eye lens is about 46% of the eye orbit height (Fig. 3, Table 8). In the front view, eyeball centre locates 11.81 ± 0.55 mm down from the topmost point of the eyeball (section #E3) and 11.80 ± 0.5 mm laterally aside from the innermost point of the eyeball (section #E4). The centre of eye lens locates 12.29 ± 0.88 mm down from the topmost point of the eyeball (section #E1) and 12.49 ± 0.78 mm laterally aside from the innermost point of the eyeball (section #E2).

**Table 5:** Eye lens positions and the ratios of orbit width/height.

Measurement section	Male (mm)	Female (mm)	Total (mm)
1	40.70 ± 1.59	39.10 ± 1.62	40.32 ± 1.73
23	23.16 ± 1.82	22.04 ± 1.56	22.89 ± 1.82
8	36.76 ± 2.08	35.66 ± 1.57	36.50 ± 20.2
21	16.83 ± 1.58	16.67 ± 1.25	16.79 ± 1.51

Ratio	Male (%)	Female (%)	Total (%)
23/1	56.91%	56.37%	56.78%
21/8	45.79%	46.74%	46.01%

These data indicate that the centre of eye lens was located laterally aside and inferiorly down from the eyeball centre in the front view. The differences between male and female eye lens positions were not statistically significant (Fig. 3).

### Correlation analysis and simple linear regression analysis

Pearson’s correlation analysis was used to determine the correlations between the measurements. In general, the correlation coefficients were ≥ 0.6, including those for sections C2, 1, 8, 21, 22, and 23, which can be used in craniofacial reconstruction/approximation to determine the position and protrusion of the eyeball (Table 6).

**Table 6:** Correlation analysis between measurement sections for eyeball position (p < 0.01).

Measurement section	Male		Female		Total	
	L	R	L	R	L	R
C2–27	0.865	0.846	0.838	0.858	0.876	0.866
C2–3	0.842	0.813	0.822	0.810	0.853	0.831
1–23	0.717	0.661	0.615	0.493	0.718	0.658
8–21	0.758	0.718	0.458	0.485	0.709	0.666
8–22	0.674	0.627	0.694	0.630	0.689	0.650
15–27	0.898	0.906	0.904	0.904	0.909	0.914
15–33	0.887	0.887	0.883	0.886	0.893	0.896
16–27	0.849	0.852	0.882	0.880	0.884	0.887
16–33	0.849	0.852	0.862	0.855	0.864	0.868
17–27	0.836	0.802	0.691	0.792	0.832	0.832
17–33	0.836	0.802	0.656	0.777	0.818	0.812
19–27	0.875	0.875	0.827	0.863	0.880	0.886
19–33	0.854	0.857	0.829	0.832	0.862	0.865
20–27	0.885	0.886	0.880	0.898	0.893	0.898
20–33	0.869	0.854	0.880	0.885	0.878	0.870

**Figure 4:**
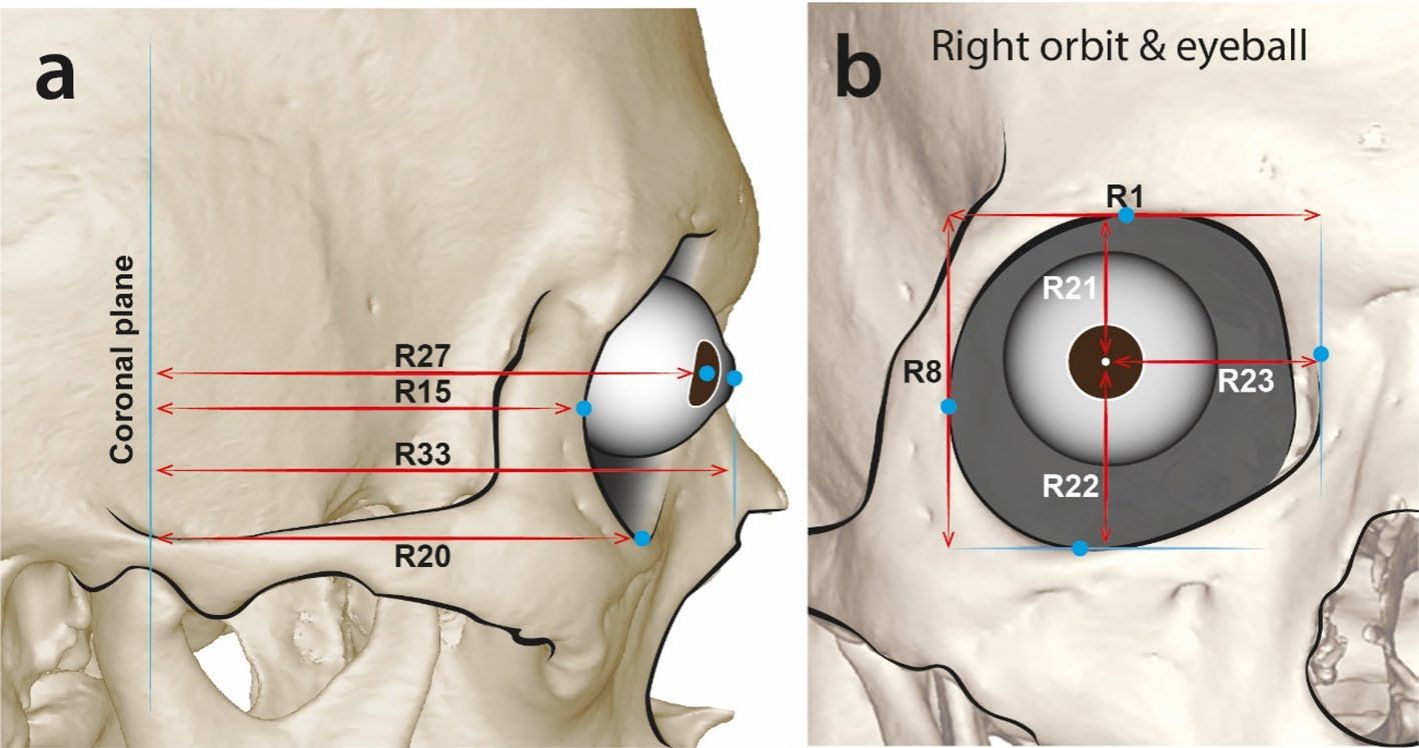
Selected measurement sections for predicting the eyeball position in the orbit in lateral view (**a**), and anterior view (**b**). R represents right side. The sections in which left-side measurements were made are identical to those of the right side.

Next, regression equations were developed by simple linear regression analysis. Some were selected to use in the craniofacial reconstruction/approximation (Tables 7, 8). The position of the eyeball in front view can be determined by the correlation between orbit height/width and eyeball centre. The protrusion of the eyeball can be determined by the correlation between the distances from the coronal plane to the landmarks on the orbit (Fig. 4).

**Table 7:** Regression equations developed from the measurments in male group.

	Regression equation	R^2^		Regression equation	R^2^
C2–L27	L27 = 0.932 $\times$ C2**–**0.312	0.748	C2–R27	R27 = 0.931 $\times$ C2 -0.479	0.716
C2–L33	L33 = 0.894 $\times$ C2 + 7.678	0.709	C2–R33	R33 = 0.893 $\times$ C2 + 7.621	0.661
**L1–L23**	**L23 = 0.844 **$\times$** L1 -11.224**	**0.515**	**R1–R23**	**R23 = 0.734** $\times$ **R1–6.687**	**0.438**
**L8–L21**	**L21 = 0.560** $\times$ **L8–3.648**	**0.575**	**R8–R21**	**R21 = 0.562** $\times$ **R8–3.923**	**0.516**
**L8–L22**	**L22 = 0.439** $\times$ **L8 + 3.662**	**0.454**	**R8–R22**	**R22 = 0.438** $\times$ **R8 + 3.939**	**0.394**
**L15–L27**	**L27 = 0.989** $\times$ **L15 + 11.550**	**0.806**	**R15–R27**	**R27 = 0.978** $\times$ **R15 + 12.421**	**0.821**
**L15–L33**	**L33 = 0.950** $\times$ **L15 + 19.126**	**0.787**	**R15–R33**	**R33 = 0.954** $\times$ **R15 + 18.983**	**0.787**
L16–L27	L27 = 1.014 $\times$ L16**–**1.316	0.748	R16–R27	R27 = 1.033 $\times$ R16–2.808	0.760
L16–L33	L33 = 0.981 $\times$ L16 + 6.290	0.721	R16–R33	R33 = 1.008 $\times$ R16 + 4.203	0.726
L17–L27	L27 = 0.781 $\times$ L17 + 50.992	0.725	R17–R27	R27 = 0.801 $\times$ R17 + 50.454	0.684
L17–L33	L33 = 0.748 $\times$ L33 + 56.568	0.700	R17–R33	R33 = 0.743 $\times$ R17 + 56.733	0.643
L19–L27	L27 = 0.950 $\times$ L19 + 4.195	0.765	R19–R27	R27 = 0.958 $\times$ R19 + 3.477	0.766
L19–L33	L33 = 0.926 $\times$ L19 + 11.095	0.729	R19–R33	R33 = 0.947 $\times$ R19 + 9.621	0.735
**L20–L27**	**L27 = 0.889** $\times$ **L20 + 11.756**	**0.782**	**R20–R27**	**R27 = 0.867 **$\times$** R20 + 13.012**	**0.786**
**L20–L33**	**L33 = 0.865** $\times$ **L20 + 18.436**	**0.755**	**R20–R33**	**R33 = 0.840** $\times$ **R20 + 19.843**	**0.730**

Bold sections were selected to be used in craniofacial reconstruction/approximation.

**Table 8:** Regression equations developed from the measurements in female group.

	Regression equation	R^2^		Regression equation	R^2^
C2–L27	L27 = 0.940 $\times$ C2–0.953	0.702	C2–R27	R27 = 0.984 $\times$ C2–4.147	0.737
C2–L33	L33 = 0.972 $\times$ C2 + 2.098	0.675	C2–R33	R33 = 1.022 $\times$ C2–2.075	0.657
**L1–L23**	**L23 = 0.619** $\times$ **L1**–**2.175**	**0.378**	**R1–R23**	**R23 = 0.449** $\times$ **R1 + 4.505**	**0.243**
**L8–L21**	**L21 = 0.349** $\times$ **L8 + 4.320**	**0.210**	**R8–R21**	**R21 = 0.407** $\times$ **R8 + 2.082**	**0.236**
**L8–L22**	**L22 = 0.652** $\times$ **L8**–**4.353**	**0.481**	**R8–R22**	**R22 = 0.593** $\times$ **R8**–**2.064**	**0.396**
**L15–L27**	**L27 = 1.007** $\times$ **L15 + 9.552**	**0.818**	**R15–R27**	**R27 = 0.951 **$\times$** R15 + 12.818**	**0.818**
**L15–L33**	**L33 = 1.005** $\times$ **L15 + 14.700**	**0.780**	**R15–R33**	**R33 = 0.973** $\times$ **R15 + 16.707**	**0.785**
L16–L27	L27 = 1.098 $\times$ L16–6.711	0.777	R16–R27	R27 = 1.019 $\times$ R16–1.534	0.774
L16–L33	L33 = 0.197 $\times$ L16–8.135	0.743	R16–R33	R33 = 1.107 $\times$ R16–2.266	0.731
L17–L27	L27 = 0.704 $\times$ L17 + 50.483	0.477	R17–R27	R27 = 0.755 $\times$ R17 + 49.414	0.627
L17–L33	L33 = 0.691 $\times$ L33 + 56.275	0.430	R17–R33	R33 = 0.799 $\times$ R33 + 53.777	0.603
L19–L27	L27 = 0.951 $\times$ L19 + 3.197	0.684	R19–R27	R27 = 0.972 $\times$ R19 + 1.816	0.745
L19–L33	L33 = 0.963 $\times$ L19 + 7.940	0.687	R19–R33	R33 = 1.014 $\times$ R19 + 4.204	0.693
**L20–L27**	**L27 = 0.969** $\times$ **L20 + 5.309**	**0.774**	**R20–R27**	**R27 = 0.905** $\times$ **R20 + 9.297**	**0.806**
**L20–L33**	**L33 = 1.028** $\times$ **L20 + 6.826**	**0.774**	**R20–R33**	**R33 = 0.985** $\times$ **R20 + 9.354**	**0.784**

Bold sections were selected to be used in craniofacial reconstruction/approximation.

In a paired-sample *t*-test comparing the differences between the measured values of the 30 sample subjects and the calculated values from the regression equations we developed in this study, all p-values for each measurement section were greater than 0.05, indicating no significant difference between the measured values and the calculated values from the regression equations (Supplementary material (out of sample validation test)).

## Discussion

In most measurement sections, male values were higher than female values and the difference of the values showed statistical significance.

In the orbital width and height, the means were generally similar to Kim et al.’s data^14^ for orbital dimensions of Korean population. In our research, the mean orbital widths were 40.72mm (R1) and 40.68mm (L1) in males and 39.24mm (R1) and 38.96mm (L1) in females and the mean orbital heights were 36.72mm (R8) and 36.80 (L8) in males and 35.60mm (R8) and 35.72mm (L8) in females. Kim’s et al. (2016) reported that orbital widths were 42.1mm in males and 40.3mm in females and orbital heights were 38.1mm in males, 37.9mm in females.

The centre of eye lens was 16.97 ± 1.62 mm in males and 16.78 ± 1.19 mm in females away from the topmost point (supraorbitale) of the orbit and 23.12 ± 1.82 mm in males and 21.95 ± 1.66 mm in females away from the innermost point (medial orbit) of the orbit. In terms of ratios, the longitudinal value was 46% of the orbit height from the superior to the inferior and the horizontal value was 57% of the orbit width from the medial to the lateral. These data indicate that the eyeballs are located rather superolaterally in the anterior view of the eye orbit.

These results show the similar patterns as Stephan et al.^15,16^ and Guyomarc’h et al.’s^10^ reports on other ethnic groups than Korean; Stephan et al. reported the distance from the topmost point (supraorbitale) to the cornea centre in front view was 16.9 mm and from the innermost point (medial orbit) of the orbit to the cornea centre in anterior view was 20.9 mm. They also reported that the eyeball takes a more superolateral position in the eye orbit in anterior view. Guyomac’h et al. reported that the distance from the centre of eye lens to the topmost point is 44.1% of the eye orbit height and from the innermost point to the eye lens centre is 57.6% of the eye orbit width, which is also indicating the superolateral position of the eyeball in the anterior view of the eye orbit^17^.

In contrast, Kim et al. reported in previous study on Koreans that the position of the eyeball, unlike other studies, was located inferolaterally^14^. It was discussed that the difference comes from the subjects’ condition; Stephan et al. researched the cadavers as sample subjects whereas Kim et al. used living subjects for the research. Guyomarc’h et al. used living subjects in supine position^10^ whereas the alive Korean subjects in Kim et al.’s were scanned in sitting upright position by CBCT (cone-beam computed tomography)^14^.

The position of the eyeball in the eye orbit has been somewhat consistent among studies but it is necessary to maintain uniform research conditions for a more accurate comparison.

In our study, the thickness of the eye lens increased with age from 3.82 to 4.40 mm, consistent pattern with the previous studies; Klein et al. reported that the eye lens thickness systematically increases with age^18^. Kim et al. reported that the eye lens thickness systematically increased from 3.56 to 4.55 mm by aging^14^.

In the eyeball diameter, the vertical diameter (E5) of eyeball was 23.68 ± 1.18 mm in males and 23.42 ± 0.73 mm in females and the horizontal diameter (E6) of eyeball was 23.64 ± 1.14 mm in males and 23.45 ± 1.17 mm in females, which is showing approximate values from the other studies on other ethnic groups. Bekerman et al. reported that emmetropic human adult eyeball have 23.7mm of vertical diameter and 24.2 mm of horizontal diameter without significant differences in different sex and age groups^19^. In Guyomac’h’s report, the average diameters are 24.6 mm in vertical, 24.3 mm in horizontal and 23.7 mm in anterior–posterior although the males’ are significantly larger^10^.

While this study identified similarities with Stephan et al. and Guyomarc’h et al.’s across population groups, such as the position of the eyeballs in front view, in Kim et al.’s study targeting Koreans different results were observed. Moreover, protrusion of the eyeballs in the eye socket has yet not been compared across the population groups. Therefore, until the accurate characteristics of eyes in faces based on particular population group are understood and can be utilized in facial reconstruction/approximation, further studies targeting particular population groups are still necessary.

We used correlation analysis of anthropometric measurements to determine the extrusion and position of the eyeball in the eye orbit. This approach is expected to provide more reliable information for the eye region of recreated face images. However, while the eye region has been reported to be the most informative area for distinguishing among faces^5^, fixation patterns differ by cultural background. For example, Europeans mainly observe the region and partially mouth whereas East Asians, including Koreans, tend to observe more on the central region of the face^9,20^. This cultural difference could lead to different results in terms of face recognition for forensic analysis, as people from different cultural background may be involved in the process of forensic identification as witnesses, law enforcement members or any other observers.

Face recognition is generally believed to be related to defining differences in relative size and position of facial features within the face^21,22^. Hence, not only the interocular distance but the distances between the other facial features must also be estimated from an unidentified skull. To this end, we incorporated facial landmarks and reference planes produced in the uniform mechanism with previous studies of correlations between eyebrows/orbits and the nose/nasal aperture groove^11,12^. This allowed us to reconstruct/approximate facial features based on the morphology of the unidentified skull as well as to minimize interference caused by estimations based on the features of different cultures.

These methods have been utilized in the craniofacial reconstruction/approximation in the National Forensic Service (NFS) for the identification of unknown human dead bodies and contributed increasing accuracy of the predicted faces. We are currently investigating the relevant features of the mouth and ears; once these studies are completed, an estimation method for the entire facial feature morphology of the Korean skull will be developed and tested for craniofacial reconstruction/approximation.

### Supplementary Information

Supplementary Information 1.

Supplementary Information 2.

Supplementary Information 3.

Supplementary Information 4.

## Data Availability

The datasets generated during and/or analysed during the current study (expect CT images taken from the corpses) are available from the corresponding author on reasonable request.
