# Supplementary material for: Developing an eyeball positioning method in the eye orbit for craniofacial identification in Korean population
Source: Sci Rep. 2024 Jul 11;14:16039. doi: 10.1038/s41598-024-66833-0 (PMC11239852; doi:10.1038/s41598-024-66833-0)
Supplement: Supplementary file 4 — Supplementary Information 4. [file 41598_2024_66833_MOESM4_ESM.pdf]

Supplementary Table A

Landmarks and definitions

Landmark	Definition
Nasion	The junction of the internasal suture with the nasofrontal suture
Prosthion	The lowermost point of the intermaxillary suture between the central incisors of the maxilla.
Auriculare	On the zygomatic root, vertically above the center of the external auditory meatus
Auriculare Midpoint	The midpoint in the 3D coordinates of both AU
Orbitale	The lowest point on the orbital rim
Bregma	Where the sagittal and coronal sutures meet
Glabella	Most projecting anterior median point on lower edge of the frontal bone, on the brow ridge, in between the superciliary arches and above the nasal root.
Lateral Orbitale	Most lateral point on the orbital rim
Median Orbitale	The point where the anterior lacrimal ridge meets frontonasal suture
Supra Orbitale	The most upper point on the orbital rim
Optic Canal Point	Uppermost point of the optic nerve canal
Lens Centre	The center of gravity of the lens
Lens Anterior	The most anterior point of the lens
Lens Posterior	The most posterior point of the lens
Anterior Chamber	The most anterior point of the anterior chamber
Globe Center	The most central point of the eyeball
Globe Superior	The uppermost point of the eyeball
Globe Inferior	The lowest point of the eyeball
Globe Lateral	The most lateral point of the eyeball
Globe Medial	The most medial mole of the eyeball.
